# Proteome Analysis of Dormancy-Released Seeds of *Fraxinus mandshurica* Rupr. in Response to Re-Dehydration under Different Conditions

**DOI:** 10.3390/ijms16034713

**Published:** 2015-03-02

**Authors:** Peng Zhang, Di Liu, Hailong Shen, Yuhua Li, Yuzhe Nie

**Affiliations:** 1 State Key Laboratory of Tree Genetics and Breeding, Northeast Forestry University, Harbin 150040, China; E-Mails: zhangpeng@nefu.edu.cn (P.Z.); shenhl-cf@nefu.edu.cn (H.S.); 2 Agricultural college, Yanbian University, Yanji 133002, China; 3 College of Life Science, Daqing Bio-tech Research Institute, Northeast Forestry University, Harbin 150040, China; E-Mails: lyhshen@126.com (Y.L.); nieyuzhe@163.com (Y.N.)

**Keywords:** *Fraxinus mandshurica*, Oleacae, seed proteomics, desiccation tolerance, drought stress

## Abstract

Desiccation tolerance is the ability of orthodox seeds to achieve equilibrium with atmospheric relative humidity and to survive in this state. Understanding how orthodox seeds respond to dehydration is important for improving quality and long-term storage of seeds under low temperature and drought stress conditions. Long-term storage of seeds is an artificial situation, because in most natural situations a seed that has been shed may not remain in a desiccated state for very long, and if dormant it may undergo repeated cycles of hydration. Different types of seeds are differentially sensitive to desiccation and this directly affects long-term storage. For these reasons, many researchers are investigating loss of desiccation tolerance during orthodox seed development to understand how it is acquired. In this study, the orthodox seed proteome response of *Fraxinus mandshurica* Rupr. to dehydration (to a relative water content of 10%, which mimics seed dehydration) was investigated under four different conditions viz. 20 °C; 20 °C with silica gel; 1 °C; and 1 °C after pretreatment with Ca^2+^. Proteins from seeds dehydrated under different conditions were extracted and separated by two-dimensional difference gel electrophoresis (2D-DIGE). A total of 2919 protein spots were detected, and high-resolution 2D-DIGE indicated there were 27 differentially expressed. Seven of these were identified using MALDI TOF/TOF mass spectrometry. Inferences from bioinformatics annotations of these proteins established the possible involvement of detoxifying enzymes, transport proteins, and nucleotide metabolism enzymes in response to dehydration. Of the seven differentially abundant proteins, the amounts of six were down-regulated and one was up-regulated. Also, a putative acyl-coenzyme A oxidase of the glyoxylate cycle increased in abundance. In particular, the presence of kinesin-1, a protein important for regulation and cargo interaction, was up-regulated in seeds exposed to low temperature dehydration. Kinesin-1 is present in all major lineages, but it is rarely detected in seed desiccation tolerance of woody species. These observations provide new insight into the proteome of seeds in deep dormancy under different desiccation conditions.

## 1. Introduction

*Fraxinus mandshurica* Rupr. (fam. Oleaceae) is an important broadleaf timber tree species in the northeastern forest region of China, and it is classified as an orthodox seed species. Seed propagation is its main mechanism of breeding, and the seeds exhibit characteristics of deep dormancy. As the seeds require 8–20 months to break dormancy, this causes difficulties for nurseries to produce seedlings of this species. In a previous study, seeds were treated with various temperatures using indoor naked stratification, and a reliable method was developed to break seed dormancy by controlling relative water content (RWC). Previously, the effects of dehydration under different conditions on the germination physiology of dormancy-released *F. mandshurica* seeds were studied [1]. It was found that the germination ability of dormancy-released *F. mandshurica* seeds was normal under low temperature conditions (1 °C), but germination ability was reduced significantly for seeds that had undergone dehydration in low temperature conditions prior to the germination experiments [1]. This interesting phenomenon is associated with seed desiccation tolerance against a variety of stresses that cause water deficits in seed cells, particularly in orthodox seeds. In this condition, it is highly likely that seeds are not really tolerant of 10% RWC (dehydration) and they will probably not survive. Still, little is known concerning the molecular mechanisms of drought stress in dormancy-released seeds of *F. mandshurica* following dehydration under different conditions. A greater understanding of desiccation tolerance could enable improvements in seed quality and storage management.

Desiccation tolerance is the ability of an organism to reach equilibrium with dry air and to resume normal metabolic functions upon rehydration [2,3,4]. Desiccation tolerance in seeds is of particular importance because the ability of seeds of many species to survive severe dehydration has been exploited to store seeds for relatively long periods. Studies of such seeds have been used to understand desiccation tolerance and “omics” approaches have been employed on these test materials [5]. Proteomic, transcriptomic and metabolomic studies have been performed to investigate seed germination and development, and such studies have largely focused on seed filling [6,7,8,9], germination and regulation of dormancy [10,11] as well as seed tolerance to abiotic stresses [12,13]. However, in data from these earlier studies, it is difficult to identify which of the observed changes are associated specifically with desiccation tolerance, as there is interference from various developmental processes that occur concomitantly. Interpretation is further complicated by the fact that desiccation tolerance is gained and lost sequentially in different parts of developing and germinating seeds. Thus, there is a need to design physiological models that uncouple desiccation tolerance from other developmental processes, but that allow investigation by “omics” methods [14,15,16]. The advantage of such an approach is the ability to follow in detail the kinetics of changes associated with the induction of desiccation tolerance [15].

Proteomics is an analysis strategy that can provide information on biochemical processes that occur in response to complex events. In addition, some proteins are modified by post-translational modification [17,18], and such post-translational modifications can be important for regulating protein function. For example, proteomics has been used to investigate cold stress responses in plants [19]. Despite increasing interest in plant proteomics, the proteomes of seeds from woody plants have been studied only rarely; however, it is important to understand how the proteome of seeds from woody plants changes during germination [20,21,22,23,24]. Changes in the proteome profiles during maturation of somatic embryos and somatic embryogenesis of woody species have been reported [25,26,27], but there are no comparative analyses of protein changes in dormancy-released *F. mandshurica* seeds after dehydration under different conditions.

There is an interest in determining the molecular mechanisms that operate in dormancy-released *F. mandshurica* seeds under different dehydration conditions. This study was thus aimed to identify the different proteins that accumulate or decline in abundance in response to different dehydration conditions, with a view to understanding the molecular and physiological mechanisms underlying desiccation tolerance in this species. Thus, in the present study, alterations in the proteome were investigated in response to desiccation (10% RWC) in dormancy-released *F. mandshurica* seeds under different dehydration conditions. The proteome was studied using two-dimensional difference gel electrophoresis (2D-DIGE) and proteins were identified by matrix-assisted laser desorption/ionization time of flight (MALDI TOF/TOF) mass spectrometry (MS).

## 2. Results and Discussion

**Figure 1:**
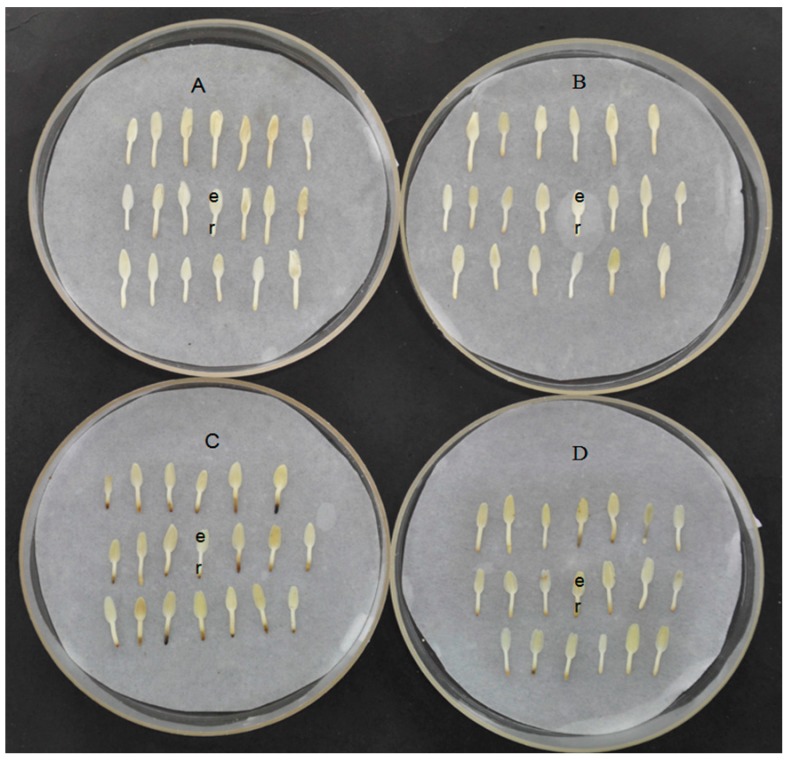
Morphology of dormancy-released seeds of *F. mandshurica* in response to re-dehydration under different conditions: (**A**) 20 °C; (**B**) 20 °C with silica gel; (**C**) 1 °C; and (**D**) 1 °C after pretreatment with Ca^2+^. Seed coat and endosperm was removed and intact embryos can be observed to contain cotyledons (e) and radicle (r).

Desiccation tolerance is a basic characteristic of most seeds, and it is thus important to understand seed survival in a desiccated state and the strategies that seeds employ to deal with water deficit. In preliminary research, it was determined that the germination ability of dormancy-released *F. mandshurica* seeds was significantly different after dehydration at 20 °C compared with 1 °C; the top of the radicle of the seeds was harmed, but the seeds retained the ability to germinate. However, the germination ability of the seeds did not differ significantly after dehydration at 20 °C compared with 20 °C supplemented with silica gel (Figure 1).

2.1. 2D-DIGE Protein Expression Analysis

**Figure 2:**
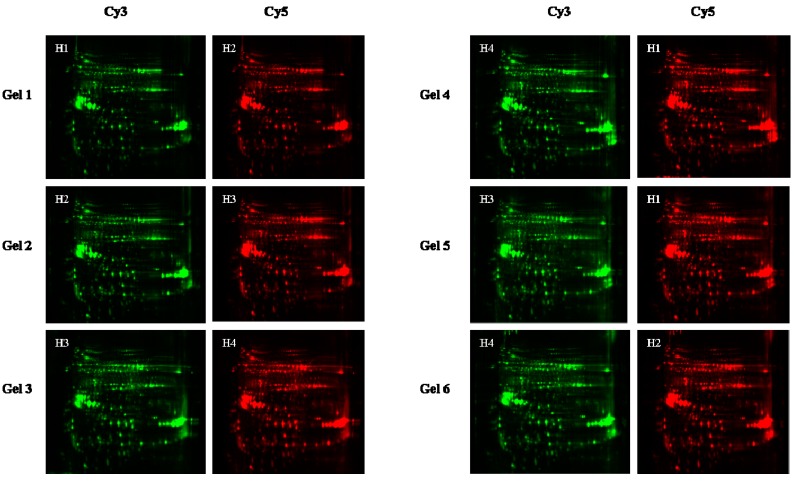
Two-dimensional difference gel electrophoresis (2D-DIGE) gel images for *F. mandshurica* seed multiplexed combinations. The high-resolution grayscale 2D-DIGE images used in the proteome analysis were generated using the Ettan DIGE Imager™ for both Cy3- and Cy5-specific exposures. Cy2-labeled internal standard, Gel 1, Gel 2, Gel 3, Gel 4, Gel 5 and Gel 6 represent the Cy dye-swap derived proteomes of the three biological replicates. H1, 1 °C sample; H2, 1 °C with Ca^2+^ sample; H3, 20 °C sample; and H4, 20 °C with silica gel sample.

**Figure 3:**
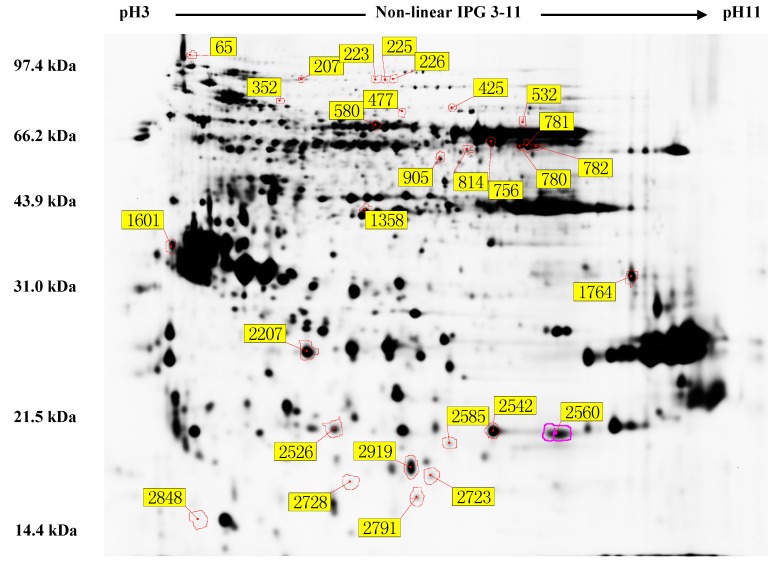
High-resolution 2D-DIGE gel reference map of the differential proteome of *F. mandshurica* seeds in response to different treatments. A representative image of a 2D-DIGE gel showing the differential protein spots determined using DeCyder™ image analysis software. Each spot number and boundary was assigned by the DeCyder™ image analysis software. Isoelectric point (PI) values are also shown.

To detect differentially abundant proteins in response to different treatments, extractable seed proteins were obtained from each of the four sample groups (20 °C; 20 °C with silica gel; 1 °C; and 1 °C after pretreatment with Ca^2+^) in biological triplicates. 2D-gel images derived from the samples are shown in Figure 2. By the use of the CyDye labeling method, approximately 2919 protein spots from the *F. mandshurica* seeds could be detected. Comparative image analysis established 27 spots that showed differential expression across the four treatment groups (Figure 3).

Image and spot analyses identified seven protein spots that exhibited significantly different abundance (up- or down-regulated) in the 10% RWC seed samples compared with seeds exposed to the desiccation treatments. These protein spots are highlighted in Figure 3 and each was assigned a numerical designation by the DeCyder Differential Analysis software. Quantification of spot volume, the use of three biological replicates and a Cy2-labeled internal standard, enabled the selection of 27 protein targets on the basis of significant differences in abundance between the four treatments (*p* < 0.05). The seven spots resulted in 27 high-confidence assignments and these data are presented in Table 1. They are catalase isozyme 3 (spots 756, 781, 782), catalase isozyme 1 (spot 780), putative acyl-coenzyme A oxidase (spot 226), and kinesin-1 (spot 1601), nucleoside diphosphate kinase (spot 2919). It is significant that a differential expression of kinesin-1 (spot 1601) between 20 °C and 1 °C treatments was detected, as it is a transport-associated protein. Six proteins were found to be associated with oxidative metabolism between the 20 °C (with silica gel) treatment group and the 1 °C (with Ca^2+^ group), specifically spots 781, 780, 756, 782, 2919 and 226. The proteins (spots 781, 780, 756, 782, 2919) in both 20 and 1 °C with Ca^2+^ treatment groups were detected. Quantitative analysis of the differential accumulation patterns of proteins from the four treatment groups showed that there was no difference in the expression of these proteins between the 20 and 20 °C with silica gel groups, and between the 1 and 1 °C after pretreatment with Ca^2+^ groups. This was expected as prior results showed that germination of seeds at 20 °C was 88% and 81% under natural drying and silica gel drying conditions, respectively [1]. Thus, seed germination was not significantly different between natural dehydration at 20 °C compared to drying at 20 °C in the presence of silica gel, which is consistent with the results of this present study. Much of the earlier work with regard to the effect of desiccation tolerance has centered on the speed of dehydration [28,29]; slow drying can significantly increase the desiccation tolerance of orthodox seeds, as this provides sufficient time for plant tissues to induce acquisition of desiccation tolerance.

The absence of differentially abundant proteins between the 1 and 1 °C after pretreatment with Ca^2+^ groups was somewhat unexpected. Ca^2+^ is a second messenger that plays an important role in abiotic stress signal transduction pathways. Cytosolic Ca^2+^ is regulated in response to exogenous stimuli, and calcium-dependent protein kinases have been identified in plants [30]. The involvement of Ca^2+^ in low temperature signaling can be inferred indirectly from observed transient changes of cytosolic Ca^2+^ in response to cold treatment [31]. Addition of exogenous Ca^2+^ can enhance abiotic stress adaptability of plants and reduce the incidence of desiccation injury, but not differentially abundant proteins between the 1 and 1 °C with Ca^2+^ groups were detected.

**Table 1:** Data for identified protein spots. Identification was performed by searching the *Viridiplantae* protein index of the non-redundant SwissProt database (ftp://ftp.ebi.ac.uk/pub/databases/uniprot/knowledgebase/uniprot_sprot.fasta.gz) using peptide mass data from MALDI TOF/TOF MS. The accession numbers and corresponding MASCOT (Software from Matrix Science, www.matrixscience.com, London, UK) scores are listed below. Theoretical values for molecular mass (*M*w) and isoelectric point (PI) were calculated using EXPASY tools.

Spot No.	Protein Name	Species	Accession No.	Protein *M*w	Protein PI	Protein Score C.I. %
226	Putative acyl-coenzyme A oxidase 3.2, peroxisomal	*Arabidopsis thaliana*	sp|Q9LMI7|ACO32_ARATH	76,479	7.93	86.829
756	Catalase isozyme 3	*Nicotiana plumbaginifolia*	sp|P49317|CATA3_NICPL	57,493.5	6.78	100.000
780	Catalase isozyme 1	*Ricinus communis*	sp|Q01297|CATA1_RICCO	56,941.5	7.08	100.000
781	Catalase isozyme 3	*Nicotiana plumbaginifolia*	sp|P49317|CATA3_NICPL	57,493.5	6.78	100.000
782	Catalase isozyme 3	*Nicotiana plumbaginifolia*	sp|P49317|CATA3_NICPL	57,493.5	6.78	100.000
1601	Kinesin-1	*Arabidopsis thaliana*	sp|Q07970|ATK1_ARATH	89,676.6	6.80	89.294
2919	Nucleoside diphosphate kinase	*Capsicum annuum*	sp|Q9M7P6|NDK_CAPA	16,372.5	6.31	99.488

It is notable that a late embryogenesis abundant (LEA) protein or LEA-like protein was not found in the list of differentially abundant proteins. Previous proteomics studies aimed at identifying proteins involved in desiccation tolerance have most often found LEA proteins. However, there is some evidence that certain seed proteins, traditionally accepted as serving storage functions, might also play roles in protecting against desiccation stress. Primarily, the LEA group of proteins is restricted to seeds and their formation may be induced by abiotic stresses. Indeed, the expression patterns of 35 out of 49 LEAs have been analyzed during the desiccation-sensitive phase in response to cold and drought stress and during seed development when desiccation tolerance is acquired [32]. LEAs are thought to be involved in desiccation tolerance and this suggestion comes almost entirely from correlative evidence provided by Cuming [33]. This evidence is convincing as accumulation of LEAs is associated with both orthodox seed maturation and imposition of a variety of stresses that cause water deficits in plant cells [33]. Nevertheless, the present study provides data from only a relative short phase at a selected time point using sampling points from dormancy-released seeds re-dehydrated to 10% RWC. However, these seeds do not germinate and this may have hindered LEA protein production, thus preventing the expected consequence of the stress response. The lack of LEA proteins in the protein extract may also indicate that the intention of targeting the re-dehydration phase was achieved. Thereafter, there would be an expected accumulation of protein protectants that might mask the critical events underlying the cellular protection processes.

### 2.2. Proteome Responses of the Cellular Protective Program Associated with Different Treatments

**Figure 4:**
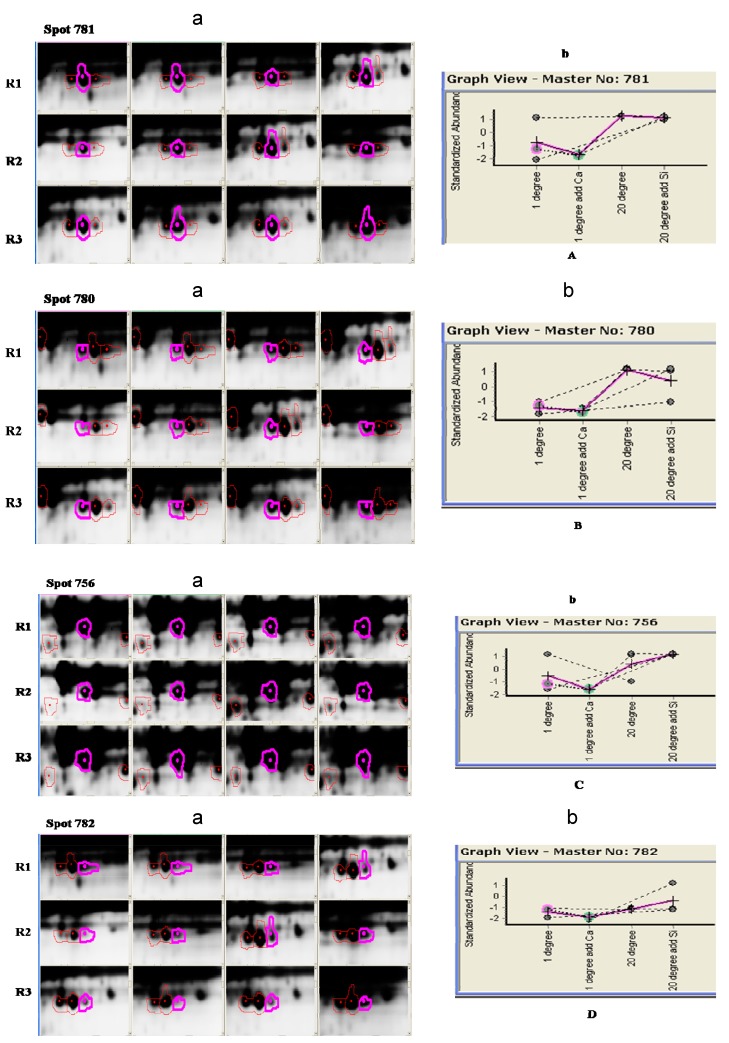

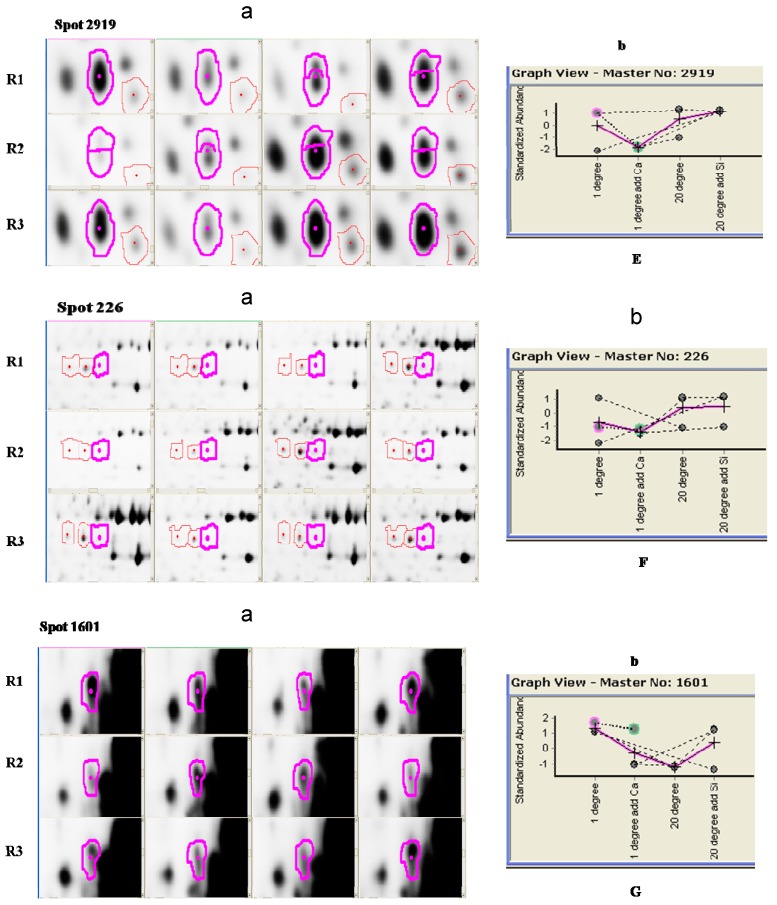
Protein spots from the seven key differential proteins (**A**–**G**) associated with the response of *F. mandshurica* seeds to dehydration to 10% RWC (relative water content) and low temperature. (**a**) 2D montages of the biological replicates of the differential proteins; (**b**) Standardized abundances of proteins **A**–**G** after different treatments.

The relatively diverse nature of the seven differentially abundant proteins, as illustrated in the detailed analysis of the key examples given in Figure 4, is indicative of the full impact on cellular function that occurs even at the relatively dehydrated level of 10% RWC. The response and adaptation mechanisms of plants to low temperature stress are complex and poorly understood. The mechanisms underlying changes at the physiological and biochemical levels have been investigated previously, especially the dynamic changes of the cytoskeleton structure and cold-stress-induced changes in the proteome [19,34]. Within this diverse set of differentially abundant proteins are those that function in particular metabolic processes, and these earlier findings provide insights into understanding the mechanisms by which cells in dormancy-released seeds of *F. mandshurica* survive desiccation under low temperature conditions.

#### 2.2.1. Changes in Energy Metabolism (Transport-Associated Proteins)

At 10% RWC, dormancy-released *F. mandshurica* seeds appear to have a metabolic focus that is aimed at transporting cellular cargo in the form of ATP and ADP. There is significant accumulation of kinesin-1 associated with the microtubule cytoskeleton, as well as up-regulation of cytoplasmic and typical scaffolding proteins that seem to link kinesin-1 to cell signaling pathways [35]. Kinesin-1 plays a critical role in the dehydration phase as it shows a large increase in expression at low temperature. It also supports microtubule movement in an ATP-dependent manner and has minus-end directed polarity, as well as having a crucial role in spindle morphogenesis in male *Arabidopsis* meiosis [36,37]. It is significant that the differential expression of kinesin-1 (spot 1601) between 20 and 1 °C treatments was detected, because this protein is often difficult to detect in seed desiccation tolerance woody plants, although members of the kinesin-1 subfamily in the kinesin superfamily are expressed at high concentrations within a cell. These proteins have been found in amoebae, plants, vertebrates and invertebrates, but have been found only rarely in plants, including *A. thaliana* [36,37,38,39,40]. Some *Arabidopsis* kinesins are associated with microtubules, Golgi stacks, vesicles, and mitochondria, and they affect organelle distribution, microtubule organization, and vesicle transport. Kinesin-1 is a dimeric motor protein that transports cellular cargo along microtubules by using the energy released from ATP hydrolysis. It is composed of three structural domains: a small globular *N*-terminus, a central α-helical coiled coil and a large globular *C*-terminus that is responsible for motor activity; it hydrolyzes ATP and binds microtubules. In the present study, a significant increase in the abundance of kinesin-1 at low temperature (1 °C) was detected. It is tempting to suggest that the protein observed in this study is somehow involved in cold stability of the microtubule. Kinesin-1 proteins may exist around the microtubules during dehydration to 10% RWC and in low temperature conditions, and it may bind directly with the microtubule and therefore play an important role in resisting or alleviating injuries caused during low temperatures and re-dehydration. Kinesin-1 may affect and regulate dynamic growth and assemblage of the microtubules, as well as providing a linkage between microtubules and other intercellular structures, thereby playing a key role in cellular morphology and adaptive strategies. There is evidence to show that cold stability (4 °C) of microtubules *in vitro* is closely related to microtubule associated proteins, and that cold stability of microtubules is improved as the ratio of microtubule associated proteins increases [41].

#### 2.2.2. Defense Mechanisms

Plants have developed a range of defense mechanisms in response to low temperature stress and dehydration. Among these is a network of enzymes involved in detoxification of excess reactive oxygen species (ROS) that accumulate during imbalance of cellular homeostasis. Six detected proteins were found to be associated with oxidative metabolism, specifically spots 781, 780, 756, 782, 2919 and 226 decreased in abundance during low temperature and dehydration conditions. The same protein, catalase, was detected from different spots on the 2D gels (781, 780, 756 and 782) and this phenomenon has been reported elsewhere [42,43,44]. This usually results from the existence of different protein isoforms, post-translational modifications or translation from alternatively spliced mRNAs. Many proteins are modified post-translationally and these changes can include phosphorylation, glycosylation and ubiquitinylation [18,45,46]. Many post-translational modifications are crucial for regulating protein function, and such changes can provide further insight important for understanding the cellular processes operating in response to different stresses. Moreover, post-translational modifications, perhaps, play an important role in mediating cellular responses to different stresses.

Significant decrease in the abundance of catalase isozyme 3 (spots 756, 782 and 781), catalase isozyme 1 (spot 780), nucleoside diphosphate kinase (NDPK; spot 2919) and putative acyl-coenzyme A oxidase (spot 226) between the 20 °C with silica gel treatment group and the 1 °C with Ca^2+^ group were detected. Catalase isozyme 3 and catalase isozyme 1 belong to the catalase family and these proteins occur in almost every aerobic organism to protect cells from toxic effects of hydrogen peroxide. A decrease in catalase activity of 50% is generally observed during chilling stress [47], but cold-acclimated (1 °C) spinach leaves show only a 25% reduction in catalase activity compared to control plants (18 °C) [48]. Catalases are mainly associated with removal of hydrogen peroxide in peroxisomes, and the major source of this hydrogen peroxide is oxidases, including acyl-CoA oxidases and glycolate oxidases. Acyl-CoA oxidases are part of the β-oxidation pathway of fatty acids, and conversion of fatty acids into carbohydrates via β-oxidation and the glyoxylate cycle occurs primarily during seed germination [49]. NDPK is a ubiquitous housekeeping enzyme that not only operates in the homeostasis of cellular nucleoside triphosphate, but this protein is also implicated in many vital cellular processes such as regulation of growth and development [50], signal transduction in response to oxidative stress, phytochrome A and mutational susceptibility [51,52,53]. Signaling and ROS-dependent up-regulation suggest an important role for NDPK2 in responses to environmental stresses associated with ROS generation [53]. AtNDPK2 has a potential role in the chilling and oxidative stress response pathways in transgenic rice plants, and genetic transformation of NDPK2 may enhance tolerance to low temperature stress [54]. Significant decreases in the abundances of the same proteins between the 20 and 1 °C with Ca^2+^ treatment groups, except for the putative acyl-coenzyme A oxidase was detected. Based on these data, it is proposed that hydrogen peroxide emitted from the putative acyl-coenzyme A oxidase during low temperature and dehydration conditions is captured by a complex consisting of catalase isozyme 1 and/or catalase isozyme 3 and NDPK. NDPK may interact with one or more catalases, thereby playing an important role in resisting damage caused by low temperatures and dehydration.

## 3. Experimental Section

### 3.1. Plant Material and Treatments

Seeds of *F. mandshurica* were collected in October 2010 from Dailing Seed Orchard in Heilongjiang Province, China. The thousand seed weight was 58.52 g and relative water content (RWC) was 8.03%. After soaking in cold H_2_O for 3 days and disinfection with 0.5% KMnO_4_ solution for 1 h, the seeds were treated with naked (without medium) stratification from 1 November 2010 to 1 May 2011 to break dormancy. Seeds were exposed to 18 °C for warm stratification for 3 months, followed by cold stratification at 5 °C for 3 months. During warm and cold stratification, the RWC of seeds were maintained at 45%–50% by adding H_2_O. After naked stratification for 6 months, the seeds had been released from dormancy. Then dormancy-released seeds were allowed to continue to dehydrate to 10% RWC under different conditions. One group of seeds was dehydrated at 20 °C (control group). And the second group of seeds was dehydrated at 1 °C. The third group of seeds was mixed with silica gel (the volume ratio of silica gel to seeds was 3:1) in a sealed box during dehydration at 20 °C. The final group of seeds was soaked in 10^−3^ M CaCl_2_ solution for 24 h at room temperature before dehydration at 1 °C. After all the seeds had been dehydrated to 10% RWC, the seed coats were removed and the seeds frozen at −80 °C. Each treatment group was performed in biological triplicate.

### 3.2. Protein Extraction

Approximately 300 mg of seeds were ground in N_2_ to a fine powder. For protein extraction, this powder was mixed with three volumes of precipitation solution containing 10% *w*/*v* CCl_3_CO_2_H (TCA) in acetone at −20 °C overnight. The solution was centrifuged for 30 min at 12,000× *g* (4 °C). The supernatant was transferred to another tube, and the precipitate was washed in an equal volume of chilled (−20 °C) acetone, before centrifugation for 30 min at 12,000× *g* (4 °C) and collection of the supernatant. This step was repeated twice and the final pellet was freeze dried. To the dry powder was added lysis buffer (7 M urea, 2 M thiourea 2 M, 4% 3-[(3-cholamidopropyl) dimethyl-ammonio]-1-propanesulfonate (CHAPS) and 30 mM Tris-HCl, pH 8.5) and this was stored at −80 °C. Prior to quantification, the pH of each protein sample was adjusted to 8.5 with concentrated HCl. Protein concentration was determined with a 2-D Quant kit (GE Healthcare, Pittsburgh, PA, USA) using bovine serum albumin (2 mg/mL) as the standard. The optimal concentration of the protein sample was 1–10 mg/mL.

### 3.3. Protein Isolation and 2D-DIGE

Each protein sample was labeled with Cy3 or Cy5 protein minimal dye (100 pMol for each 50 µg of protein), according to the manufacturer’s directions (GE Healthcare). An internal standard sample was prepared by pooling an equal protein quantity from each of the samples. Each protein sample was labeled with Cy3 or Cy5 fluorescent dye separately according to the experiment design. Thus protein mixes were prepared in the following sequence for 2D-DIGE analysis: gel 1, Cy3 1 °C replicate 1 plus Cy5 1 °C after pretreatment with Ca^2+^ replicate 1; gel 2, Cy3 1 °C after pretreatment with Ca^2+^ replicate 2 plus Cy5 20 °C replicate 1; gel 3, Cy3 20 °C replicate 2 plus Cy5 20 °C with silica gel replicate 1; gel 4, Cy3 20 °C with silica gel replicate 2 plus Cy5 1 °C replicate 2; gel 5, Cy3 20 °C replicate 3 plus Cy5 1 °C replicate 3; gel 6, Cy3 20 °C with silica gel replicate 3 plus Cy5 1 °C after pretreatment with Ca^2+^ replicate 3. Each sample contained an equal amount of a Cy2-labeled internal standard protein mix derived from pooling an equal amount of protein from each of the four extracts and this allowed for normalization of each gel. The labeling procedure was performed for the CyDye DIGE fluors (minimal dyes) and Ettan DIGE according to the manufacturer’s instructions (GE Healthcare). Prior to isoelectric focusing (IEF), labeled samples were mixed with an equal volume of 2× sample buffer (7 M urea, 2 M thiourea, 4% *w*/*v* CHAPS, and 0.004% bromophenol blue) containing 2% dithiothreitol (DTT) and 1% immobilized pH gradient (IPG) buffer (pH 3–11; GE Healthcare) to bring the final volume to 450 µL. IEF was carried out using 24-cm Immobiline Dry Strip gels (pH 3–11) at 25 °C. The electrophoresis consisted of six steps: 30 V for 12 h, step-n-hold; 100 V for 1 h, step-n-hold; 1000 V for 1 h, gradient; 8000 V for 3 h, gradient; 8000 V for 55,000 V·h, step-n-hold; and 500 V for hold, step-n-hold. After IEF, strip gels were removed and equilibrated for 15 min in 1% DTT, 6 M urea, 75 mM Tris-HCl (pH 8.8), 29.3% glycerol, 2% sodium dodecylsulfate (SDS) and 0.002% bromophenol blue. After removing from the equilibrium buffer, the strips were allowed a second equilibration for 15 min in the same equilibration buffer without DDT but this time supplemented with 2.5% iodoacetamide. The equilibrated strips were then placed on a top of an SDS-polyacrylamide gel (12.5%) on low fluorescent glass plates. Separation was performed at 25 °C on an Ettan™ DALT-six apparatus at 3 W/gel for 45 min, and then 17 W/gel until the bromophenol blue front had reached the bottom of the gel.

### 3.4. Image Acquisition and 2D-DIGE Image Analysis

CyDye-labeled gels were scanned using a Typhoon™ 9400 Trio imager (GE Healthcare) choosing parameters appropriate for the excitation and emission wavelengths of each dye (Cy2: 488/520 nm; Cy3: 532/580 nm; and Cy5: 633/670 nm). Gels were scanned individually at 100 µM resolution.

The voltage of the photo multiplier tube was adjusted for fluorescence intensity between 60,000–100,000. Images were analyzed using DeCyder Difference Analysis software version 7.0 (GE Healthcare). Scanned images were imported by Image Loder using the differential in-gel analysis module of this software, and inter-gel matching was performed using the Biological Variation Analysis module. To calculate the abundance change of each matched spot pair, one-way analysis of variance tests and paired student’s *t*-test were used, with *p* < 0.05 considered to be significant. Gels were stained with Coomassie Brilliant Blue (CBB) and spots of interest were excised from the preparative gels.

### 3.5. Identification of Proteins by MALDI TOF/TOF MS

Spots of interest were picked from preparative CBB-stained gels and de-stained in a solution containing 100 mM NH_4_HCO_3_ and 30% CH_3_CN-H_2_O (30:70, *v*/*v*) for 15 min. De-stained spots were centrifuged briefly and the supernatant was removed before placing in CH_3_CN for 5 min. Gel pieces were dried at room temperature for 5 min so that the CH_3_CN had completely evaporated, and 2 µL of 50 ng/µL trypsin (adjusted to pH 7.5–8.5 with 50 mM NH_4_HCO_3_) was added. Gel pieces were placed for 60 min at 4 °C, and then 2 µL of 50 mM NH_4_HCO_3_ (pH 7.8–8.0) were added to cover the gel pieces completely. Digestion was performed overnight at 37 °C.

For MALDI-TOF MS, each digest (1 µL) was mixed with matrix solution ((1 µL) 5 mg/mL α-cyano-4-hydroxycinnamic acid) and deposited onto the MALDI target, which was dried at room temperature. Tryptic peptides were analyzed by ABI 4800 plus MALDI TOF/TOF Analyzer. MS survey scans were acquired for the mass range *m*/*z* 800–2000 in the positive-ion mode and accumulated at a laser intensity of 3700 with an acceleration of 20 kV. Spectra were calibrated using PeptideCalibStandard before survey scans were prepared giving mass outlier errors of 5 ppm. Peaks were detected with a minimum signal/noise ratio >20. Peptide precursor ions corresponding to contaminants (including trypsin and keratin autolytic products) were excluded with a mass tolerance of ±0.5 *m*/*z*. In total, 15 precursor ions with the strongest signals were selected for the MS/MS scan. The MS/MS spectra were accumulated at a laser intensity of 4100, an acceleration of 1 kV and a pressure of 5 × 10^−8^ Torr. Mass spectra were processed using Global Server Workstation software. A database search for protein identifications was performed using SwissProt *Viridiplantae* in MS and MS/MS combination mode (see Supplementary data).

## 4. Conclusions

The sensitivity of 2D-DIGE, coupled with protein identification using MALDI TOF/TOF, has permitted the study of the dynamic proteome of dormancy-released *F. mandshurica* seeds dehydrated at a critical stage to 10% RWC and low temperature, thereby providing insight into the processes that lead to desiccation-induced damage. Kinesin-1 has been rarely found in wood seed plants, and this protein may play a role in the establishment of cell morphology and environmental adaptation strategies. Changes in proteins associated with ROS also indicate that defense mechanisms in response to low temperature stress and dehydration undergo important alterations. These results indicate that differentially abundant proteins change cellular functions in dormancy-released *F. mandshurica* seeds during low temperature and dehydration conditions, and highlight some important processes involved in cellular protection processes, transport processes and regulation. These results give a unique insight into the processes of cellular protection and transport that may be explored for improving desiccation tolerance in dormancy-released seeds of this species.

## Supplementary Materials

Supplementary materials can be found at http://www.mdpi.com/1422-0067/16/03/4713/s1.
